# *Pseudomonas aeruginosa* isolation is an important predictor for recurrent hemoptysis after bronchial artery embolization in patients with idiopathic bronchiectasis: a multicenter cohort study

**DOI:** 10.1186/s12931-023-02391-9

**Published:** 2023-03-18

**Authors:** Le-Le Wang, Hai-Wen Lu, Ling-Ling Li, Yong-Hua Gao, Yu-Hua Xu, Hong-Xiao Li, Yun-Zhu Xi, Fu-Sheng Jiang, Xue-Feng Ling, Wei Wei, Fa-Jiu Li, Bei Mao, Sen Jiang, Jin-Fu Xu

**Affiliations:** 1 Department of Respiratory and Critical Care Medicine, Shanghai Pulmonary Hospital, School of Medicine, Tongji University, Shanghai, China; 2 Institute of Respiratory Medicine, School of Medicine, Tongji University, Shanghai, China; 3 Department of Interventional Radiology, Shanghai Pulmonary Hospital, School of Medicine, Tongji University, Shanghai, China; 4 Department of Interventional Radiology, Jiangxi Chest Hospital, The Third Affiliated Hospital, Nanchang Medical College, Nanchang, China; 5 Department of Respiratory and Critical Care Medicine, The Second People’s Hospital of Jingdezhen, Jingdezhen, China; 6 Department of Respiratory and Critical Care Medicine, The Second Affiliated Hospital of Hengyang Medical School, University of South China, Hengyang, China; 7 Department of Interventional Radiology, People’s Hospital of Yichun City, YiChun, China; 8 Department of Respiratory and Critical Care Medicine, Affiliated Hospital of Jiujiang University, Jiujiang, China; 9 Department of Interventional Radiology, Jingzhou Hospital Affiliated to Yangtze University, JingZhou, China; 10 Department of Pulmonary and Critical Care Medicine, Affiliated Hospital of Jianghan University, Wuhan, China

## Abstract

**Background:**

Nearly half of bronchiectasis patients receiving bronchial artery embolization (BAE) still have recurrent hemoptysis, which may be life-threatening. Worse still, the underlying risk factors of recurrence remain unknown.

**Methods:**

A retrospective cohort was conducted of patients with idiopathic bronchiectasis who received BAE from 2015 to 2019 at eight centers. Patients were followed up for at least 24 months post BAE. Based on the outcomes of recurrent hemoptysis and recurrent severe hemoptysis, a Cox regression model was used to identify risk factors for recurrence.

**Results:**

A total of 588 individuals were included. The median follow-up period was 34.0 months (interquartile range: 24.3–53.3 months). The 1-month, 1-year, 2-year, and 5-year cumulative recurrent hemoptysis-free rates were 87.2%, 67.5%, 57.6%, and 49.4%, respectively. The following factors were relative to recurrent hemoptysis: 24-h sputum volume (hazard ratio [HR] = 1.99 [95% confidence interval [95% CI]: 1.25–3.15, *p* = 0.015]), isolation of *Pseudomonas aeruginosa* (HR = 1.50 [95% CI: 1.13–2.00, *p* = 0.003]), extensive bronchiectasis (HR = 2.00 [95% CI: 1.29–3.09, *p* = 0.002]), and aberrant bronchial arteries (AbBAs) (HR = 1.45 [95% CI: 1.09–1.93, *p* = 0.014]). The area under the receiver operating characteristic curve of the nomogram was 0.728 [95% CI: 0.688–0.769].

**Conclusions:**

Isolation of *Pseudomonas aeruginosa* is an important independent predictor of recurrent hemoptysis. The clearance of *Pseudomonas aeruginosa* might effectively reduce the hemoptysis recurrence rate.

**Supplementary Information:**

The online version contains supplementary material available at 10.1186/s12931-023-02391-9.

## Introduction

Bronchiectasis, a permanent and progressive bronchial deformation due to fibrosis of the bronchial wall muscles and destruction of elastic tissue, presents with chronic cough, purulent sputum, and recurrent hemoptysis [1, 2]. Aetiologies include congenital, idiopathic, post-infection, immunodeficiency, chronic obstructive pulmonary disease, connective tissue disease, ciliary dysfunction, and allergic bronchopulmonary aspergillosis [2, 3], with idiopathic bronchiectasis the most common in China [4, 5].

The United States Bronchiectasis Research Registry reported that 23% of patients with bronchiectasis had a history of hemoptysis [1]. The hemoptysis volume was larger than other diseases with more cases leading to recurrence [6]. In-hospital death rates due to hemoptysis among adults with bronchiectasis occurred in approximately 4.5–9.2% of hospitalizations [7, 8]. Bleeding arteries of patients with idiopathic bronchiectasis included bronchial arteries (BAs) and non-bronchial systemic arteries (NBSAs) [9]. BAs are systemic arteries running through the hilum around the main bronchus that enter the lung, including BAs directly from the descending thoracic aorta, and aberrant BAs (AbBAs) from outside this area [10].

Bronchial arterial embolization (BAE) has become the first-line treatment for hemoptysis [11]. However, nearly one-third of patients experience recurrent hemoptysis one year after BAE, and one-fifth of patients after BAE still require repeated embolization [12, 13]. A few clinical studies suggest that recurrent severe hemoptysis after BAE was associated with hemoptysis history, age, bronchial-pulmonary shunt, and NBSAs [2, 12]. However, these studies have overlooked other clinical indicators including pathogenic microorganisms and computed tomography (CT) imaging. In addition, few studies have specifically investigated the risk factors associated with recurrent hemoptysis with idiopathic bronchiectasis.

Therefore, this multicenter retrospective cohort study aimed to report the prevalence of the recurrence of hemoptysis in patients with idiopathic bronchiectasis who received BAE. Furthermore, we aimed to identify the independent risk factors of recurrence comprehensively including medical histories, pathogens, CT imaging, and responsible arteries.

## Methods

### Study patients

**Fig. 1:**
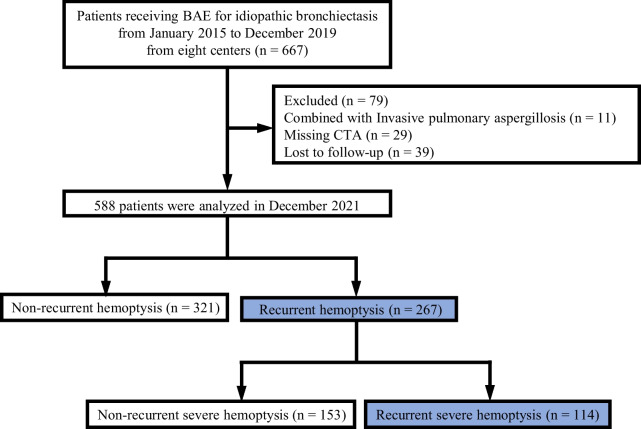
Flowchart of enrolled patients. *BAE* bronchial artery embolization, *CTA* computed tomography angiography

A total of 667 patients with idiopathic bronchiectasis treated with BAE for hemoptysis between January 1, 2015, and December 31, 2019, from eight centers were enrolled in this multicenter, retrospective, observational cohort study. The study was approved by the institutional ethics review board of eight centers. The inclusion criteria were as follows: (1) age ≥ 18 years, (2) patients were diagnosed with idiopathic bronchiectasis according to the British Thoracic Society’s guideline for bronchiectasis in 2019 [14] and the expert consensus of Chinese bronchiectasis in 2021 [15], and (3) patients underwent BAE to control hemoptysis. BAE was performed on patients with life-threatening hemoptysis, which manifested as a hemoptysis volume > 100 mL/d, abnormal gas exchange or airway obstruction, hemodynamic instability [16], or repeated hemoptysis that did not respond to medical treatment. The exclusion criteria were as follow: (1) missing CT angiography (CTA) (n = 39), (2) bronchiectasis combined with invasive pulmonary aspergillosis (n = 10), (3) technical failure (n = 1), and (4) lost to follow-up (n = 29). Based on these criteria, data from 588 patients were analyzed. A flowchart of the enrolled patients is shown in Fig. 1.

### Study design and data collection

The baseline was defined as the time of BAE. Demographic characteristics (age, sex, smoking status, and duration of bronchiectasis), clinical data (symptoms on admission, comorbidities, pathogen findings, and CT and CTA findings), and treatment regimens were collected at baseline in detail from the electronic medical records of enrolled patients. CT findings included the number of broncniectatic lobes, bronchoarterial ratio, bronchiectatic type, and the presence of atelectasis and emphysema. CTA findings included the name and number of abnormal arteries. To minimize information bias, the imaging data were evaluated by two radiologists, and the results were averaged.

Patients were followed up via telephone interviews that covered their general conditions, recurrence status, recurrence time, and management in detail. The end of follow-up was December 2021. Recurrent hemoptysis was defined as the expectoration of blood alone without mucus from the airways or lung parenchyma after BAE [17]. Recurrent severe hemoptysis was defined as hemoptysis requiring repeated BAE, lobectomy, or death from hemoptysis after BAE [18]. Recurrence-free time was calculated from the date of BAE to the date of recurrence, death, or last follow-up.

### Bronchial arterial angiography and embolization

BAE was performed by trained interventional doctors at all eight centers. All doctors received the same treatment procedure training [19] by designated radiologists of Shanghai Pulmonary Hospital, the special pulmonary vascular interventional center in China that experience with about 10,000 cases with BAE and more than 1000 cases per year. First, CTA (slice thickness, 0.625 or 1 mm; slice gap, 0.625 or 1 mm) was performed prior to BAE to assess lung lesions and possible relevant arteries [18]. Second, angiography was performed through various types of 4F or 5F catheters based on the CTA findings through the femoral artery to confirm relevant arteries. Abnormal angiographic signs were vascular proliferation and distortion with extravasation of contrast medium in lung lesions, or systemic arterial-pulmonary circulation shunts. Lastly, all confirmed relevant arteries underwent super-selective catheterization and embolization with a coaxial microcatheter system. The embolic materials were 300–700 µm polyvinyl alcohol particles. Successful embolization was defined as no obvious distal vascular signs during the second angiography.

### Statistical analysis

Continuous variables, if conforming to a normal distribution, were expressed as the mean ± standard deviation (SD), and if not conforming to a normal distribution, were expressed as median (interquartile range). Categorical variables were presented as numbers (percentages). To compare the difference between groups, we used the t-test for normal distribution continuous variables, the Mann–Whitney *U* test for non-normal distribution continuous variables, and the χ^2^ and Fisher exact test for categorical variables. Survival curves were plotted by the Kaplan–Meier method and compared by the log-rank test. Factors with a *p*-value < 0.05 of univariate regression and clinical relevance were included in the multivariate regression model. Significant predictors in multivariate Cox regression were used to construct a nomogram model. The model discrimination ability was evaluated by calculating the area under the receiver operating characteristic curve (AUC), while the calibration plot was used to graphically assess the calibration. A *p*-value < 0.05 was considered statistically significant. All of the analyses were conducted using SPSS (version 25), R software (version 4.1.0), and GraphPad Prism (version 8.3.0).

## Results

### Baseline characteristics

The features of all 588 patients are summarized in Table 1. The mean age was 59.9 years (range, 18–93), and 276 patients (46.9%) were female. No severe procedure-related complications occurred. Minor complications included chest tightness (n = 36), chest pain (n = 33), hematoma at the puncture site (n = 11), fever (n = 7), and dysphagia (n = 5) (Table 1). All symptoms were relieved spontaneously or after appropriate treatment (Additional file 2: Fig. S2).

**Table 1:** Comparison of patient characteristics

Category	All patients (N = 588)	Recurrent hemoptysis			Recurrent severe hemoptysis		
		No (N = 321)	Yes (N = 267)	*p* value	No (N = 474)	Yes (N = 114)	*p* value
Age, years	59.9 ± 12.6	59.8 ± 12.5	59.9 ± 12.7	0.899	59.2 ± 12.7	62.4 ± 11.9	0.016
Sex (female)	276 (46.9)	157 (48.9)	119 (44.6)	0.294	218 (46.0)	58 (50.9)	0.348
Body mass index, kg m^−2^	21.6 ± 2.8	21.4 ± 3.4	21.4 ± 3.4	0.608	21.6 ± 3.1	21.3 ± 3.1	0.405
Smoking							
Non-smoker	392 (66.7)	203 (62.9)	189 (71.2)	0.071	320 (66.9)	72 (65.5)	0.345
Ex-smoker	143 (24.3)	84 (26.2)	59 (22.1)		112 (23.4)	31 (28.2)	
Current smoker	53 (9.0)	35 (10.9)	18 (6.7)		46 (9.6)	7 (6.4)	
Duration of bronchiectasis, years	10 (5.20)	10 (4.20)	20 (8.30)	< 0.001	10 (5.20)	20 (10.30)	< 0.001
Symptoms							
Duration of hemoptysis, years	7 (2.20)	5 (1.12)	10 (2.20)	0.001	5 (1.20)	10 (3.30)	0.001
Hemoptysis volume, mL	180 (150.210)	180 (150.200)	200 (150.220)	0.078	180 (140.203)	200 (158.235)	0.013
24-h sputum volume†							
Minimal	139 (23.6)	97 (30.2)	42 (15.7)	< 0.001	123 (25.9)	16 (14.0)	0.005
Few	225 (38.3)	126 (39.3)	99 (37.1)		182 (38.4)	43 (37.7)	
Medium	159 (27.0)	77 (24.0)	82 (30.7)		125 (26.4)	34 (29.8)	
Massive	65 (11.1)	21 (6.5)	44 (16.5)		44 (9.3)	21 (18.4)	
Hemoptysis related factors							
Hypertension	130 (22.1)	69 (21.5)	61 (22.8)	0.694	106 (22.4)	24 (21.1)	0.762
Thrombocytopenia	19 (3.2)	10 (3.1)	9 (3.4)	0.862	15 (3.2)	4 (3.5)	0.773
Anticoagulant	22 (3.7)	8 (2.5)	14 (5.2)	0.080	17 (3.6)	5 (4.4)	0.783
Pathogen findings							
Isolation of *Pseudomonas aeruginosa*	140 (23.8)	44 (13.7)	96 (36.1)	< 0.001	84 (17.7)	56 (49.1)	< 0.001
Isolation of other pathogenic microorganisms*	23 (3.9)	10 (3.1)	13 (4.9)	0.275	21 (4.4)	2 (1.8)	0.281
Radiographic findings							
Number of bronchiectatic lobes ≥ 3	427 (72.6)	196 (61.1)	231 (86.5)	< 0.001	329 (69.4)	98 (86.0)	< 0.001
Bronchoarterial ratio^§^							
1–2 times	261 (44.4)	176 (54.8)	85 (31.8)	< 0.001	232 (48.9)	29 (25.4)	< 0.001
2–3times	110 (18.7)	62 (19.3)	48 (18.0)		87 (18.4)	23 (20.2)	
> 3times	217 (36.9)	83 (25.9)	134 (50.2)		155 (32.7)	62 (54.4)	
Bronchiectatic type							
Cylindrical	161 (27.4)	113 (35.1)	48 (18.0)	< 0.001	142 (30.0)	19 (16.7)	0.001
Cystic	91 (15.5)	59 (18.3)	32 (12.0)		79 (16.7)	12 (10.5)	
Mixed	336 (57.1)	149 (46.4)	187 (70.0)		253 (53.4)	83 (72.8)	
Emphysema	115 (19.6)	75 (23.4)	40 (15.0)	0.011	96 (20.3)	19 (16.7)	0.386
Atelectasis	161 (27.4)	79 (24.6)	82 (30.7)	0.099	118 (24.9)	43 (37.7)	0.006
Complications after BAE							
Chest tightness	36 (6.1)	19 (5.9)	17 (6.4)	0.188	26 (5.5)	10 (8.8)	0.753
Chest pain	33 (5.6)	17 (5.3)	16 (6.0)		26 (5.5)	7 (6.1)	
Hematoma at the puncture site	11 (1.9)	5 (1.6)	6 (2.2)		9 (1.9)	2 (1.8)	
Fever	7 (1.2)	6 (1.9)	1 (0.4)		6 (1.3)	1 (0.9)	
Dysphagia	5 (0.9)	5 (1.6)	0 (0)		5 (1.1)	0 (0)	

Data are n (%), mean ± SD, or median (IQR). *BAE* bronchial artery embolization

^†^Minimal, few, medium, and massive represented sputum volumes of < 10 mL, 10–50 mL, 50–100 mL, and ≥ 100 mL, respectively

*Other pathogenic microorganisms including *Klebsiella pneumoniae, Haemophilus, Acinetobacter baumannii,* and *Stenotrophomonas maltophilia*

^§^The ratio of bronchia lumen to vessel diameter

The median duration of bronchiectasis before BAE was 10 years (interquartile range [IQR], 5–20 years), while the median duration of hemoptysis before BAE was 7 years (IQR, 2–20 years). Hypertension was present in 130 patients (22.1%). The pathogen with the highest isolation rate in sputum was *Pseudomonas aeruginosa* (*P. aeruginosa*), accounting for 140 patients (23.8%). Other bacteria isolated from sputum included *Klebsiella pneumoniae, Haemophilus, Acinetobacter baumannii,* and *Stenotrophomonas maltophilia*. The bronchoarterial ratio was more than three times in 217 patients (36.9%). Three or more bronchiectatic lobes were presented in 427 patients (72.6%), and 336 patients (57.1%) had mixed bronchiectasis (Table 1).

### Outcomes of post-BAE

**Fig. 2:**
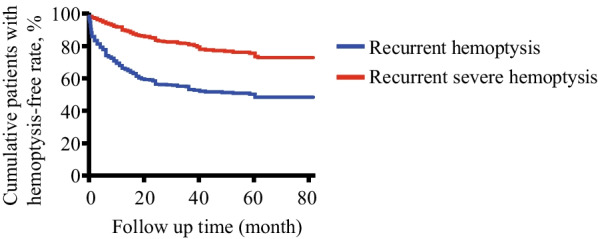
The cumulative hemoptysis control rate for all patients (n = 588). The median follow-up period of recurrence hemoptysis was 34.0 months (IQR, 24.3–53.3 months; range, 0.1–81.1 months). Hemoptysis occurred in 267 (45.4%) of 588 patients after BAE. The 1-month, 1-year, 2-year, and 5-year cumulative recurrence-free rates were 87.2%, 67.5%, 57.6%, and 49.4%, respectively. A total of 114 patients (19.4%) experienced recurrent severe hemoptysis, including 6 cases of pulmonary lobectomy and 9 cases of death. The 1-year, 2-year, and 5-year cumulative exacerbated hemoptysis-free rates were 91.4%, 84.9%, and 74.8%, respectively

The median follow-up period of recurrent hemoptysis was 34.0 months (IQR, 24.3–53.3 months; range, 0.1–81.1 months). Hemoptysis occurred in 267 patients (45.4%) after BAE. The 1-month, 1-year, 2-year, and 5-year cumulative recurrent hemoptysis-free rates were 87.2%, 67.5%, 57.6%, and 49.4%, respectively. A total of 114 patients (19.4%) experienced recurrent severe hemoptysis, including 6 cases of pulmonary lobectomy, and 9 cases of death. The 1-year, 2-year, and 5-year cumulative recurrent severe hemoptysis-free rates were 91.4%, 84.9%, and 74.8%, respectively (Fig. 2).

Massive sputum quantities (18.4% [21/114] vs. 9.3% [44/474]; *p* < 0.001), isolation of *P. aeruginosa* (49.1% [56/114] vs. 17.7% [84/474]; *p* < 0.001), three or more lobes affected (86.0% [98/114] vs. 69.4% [329/474]; *p* = 0.001), more than two times the bronchoarterial ratio (54.4% [62/114] vs. 32.7% [155/474]; *p* < 0.001), mixed bronchiectatic type (72.8% [83/114] vs. 53.4% [253/474]; *p* < 0.001) (Table 1), and abnormal AbBAs (31.8% [35/114] vs. 18.6% [89/474]; *p* = 0.002) were more prevalent in the recurrent severe hemoptysis group (Table 2).

**Table 2:** Comparison of abnormal arteries

Arteries	All patients (N = 588)	Recurrent hemoptysis			Recurrent severe hemoptysis		
		No (N = 321)	Yes (N = 267)	*p* value	No (N = 474)	Yes (N = 114)	*p* value
Left BAs	374 (63.6)	216 (67.1)	158 (59.4)	0.054	310 (64.9)	64 (58.2)	0.190
Right intercostobronchial trunk	366 (62.6)	199 (61.8)	167 (62.8)	0.807	298 (62.3)	68 (61.8)	0.918
Common BAs	268 (45.6)	145 (45.0)	123 (46.2)	0.769	221 (46.2)	47 (42.7)	0.505
Right BAs	126 (21.4)	69 (21.4)	57 (21.4)	1.000	104 (21.8)	22 (2.0)	0.685
Left intercostobronchial trunk	16 (2.7)	5 (1.6)	11 (4.1)	0.055	12 (2.5)	4 (3.6)	0.742
Internal thoracic arteries	202 (34.4)	106 (32.9)	96 (36.1)	0.420	167 (34.9)	35 (31.8)	0.535
Costocervical trunk	41 (7.0)	25 (7.8)	16 (6.0)	0.407	35 (7.3)	6 (5.5)	0.488
Lateral thoracic arteries	50 (8.5)	27 (8.4)	23 (8.6)	0.910	37 (7.7)	13 (11.8)	0.167
Thyrocervical trunk	24 (4.1)	11 (3.4)	13 (4.9)	0.37	18 (3.8)	6 (5.5)	0.589
Subscapular arteries	14 (2.4)	8 (2.5)	6 (2.3)	0.856	9 (1.9)	5 (4.5)	0.192
Proper esophageal arteries	183 (31.1)	88 (27.3)	95 (35.7)	0.029	145 (3.3)	38 (34.5)	0.390
Intercostal arteries	68 (11.6)	38 (11.8)	30 (11.3)	0.844	52 (1.9)	16 (14.5)	0.278
Inferior phrenic arteries	277 (41.1)	139 (43.2)	138 (51.9)	0.035	217 (45.4)	60 (54.5)	0.083
Left gastric arteries	10 (1.7)	3 (0.9)	7 (2.6)	0.205	6 (1.3)	4 (3.6)	0.183
Abnormal AbBAs on CTA	124 (21.1)	49 (15.2)	75 (28.2)	< 0.001	89 (18.6)	35 (31.8)	0.002

Results are expressed as number (percentage). AbBAs, aberrant BAs; CTA, Computed tomography angiography

### Independent predictors of recurrent hemoptysis

**Fig. 3:**
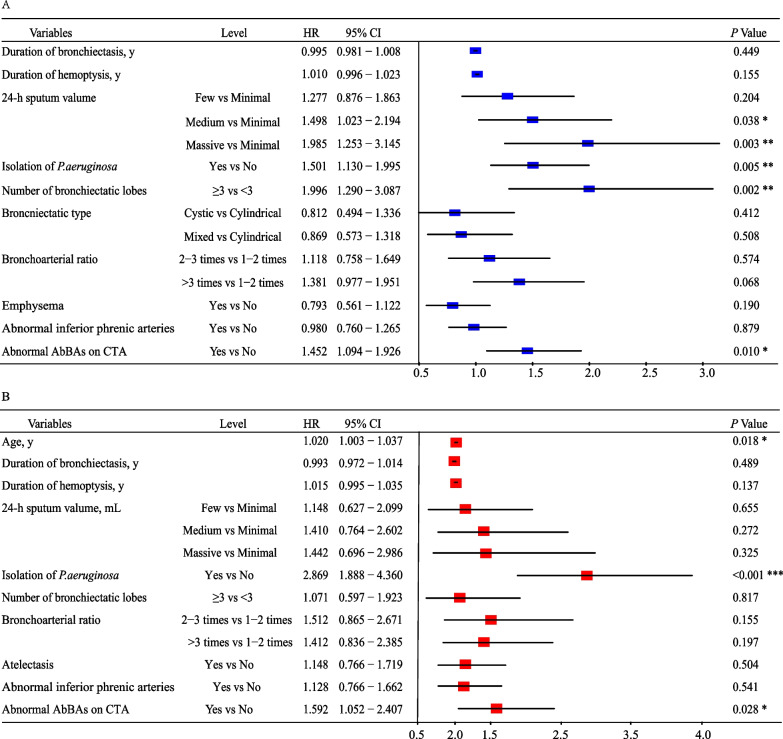
The multivariate Cox proportional hazards regression of recurrent hemoptysis. **A** Risk factors of recurrent hemoptysis in the model. **B** Risk factors of recurrent severe hemoptysis in the model. The figure presents the HRs and the 95% CI. *AbBAs* aberrant bronchial arteries, *CTA* computed tomography angiography

The results of the univariate Cox regression analysis are shown (Additional file 4: Tables S1 and S2). The independent predictors of recurrent hemoptysis were 24-h sputum volume (hazard ratio [HR], 1.99; 95% CI, 1.25–3.15), isolation of *P. aeruginosa* (HR, 1.50; 95% CI, 1.13–2.00), extensive bronchiectasis (HR, 2.00; 95% CI, 1.29–3.09), and abnormal AbBAs (HR, 1.45; 95% CI, 1.09–1.93) (Fig. 3A) The independent factors associated with recurrent severe hemoptysis were age (HR,1.02; 95% CI, 1.00–1.04), the isolation of *P. aeruginosa* (HR, 2.87; 95% CI, 1.89–4.36), and AbBAs (HR, 1.59; 95% CI, 1.05–2.41) (Fig. 3B). Recurrent hemoptysis was present in 96 of 140 patients (68.6%) in the isolation of *P. aeruginosa* group, 75 of 124 patients (60.5%) in the AbBAs group, 231 of 427 patients (54.1%) in the three or more lobes affected group, and 44 of 65 patients (67.7%) with massive sputum production. The impact of predictors on recurrence was confirmed by Kaplan–Meier curve analysis. The hemoptysis control rates were significantly lower in the isolation of *P. aeruginosa* group (*p* < 0.001), AbBAs group (*p* < 0.001), three or more lobes affected group (*p* < 0.001), and massive 24-h sputum production group (*p* < 0.001) (Additional file 1: Figure S1).

**Fig. 4:**
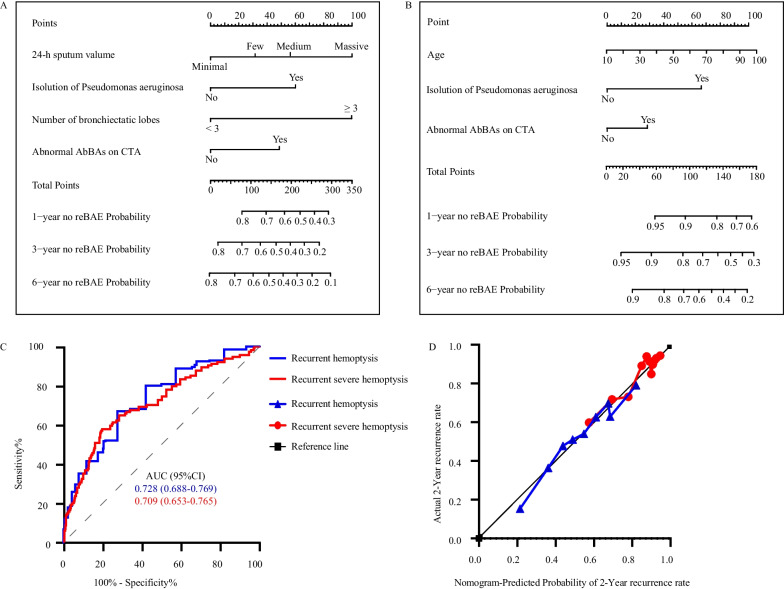
Predict recurrent of idiopathic bronchiectasis-related hemoptysis after BAE. **A** Prognostic nomograms to Predict recurrent hemoptysis. **B** Prognostic nomograms to Predict recurrent severe hemoptysis. **C** Receiver operating characteristic curves and areas under the curve (AUC) were used to determine the overall predictive value of recurrence hemoptysis or recurrent severe hemoptysis. An AUC value of 0.5 was considered the reference value and was expressed as a grey dotted line. **D** The calibration plot of the nomogram for predicting 2 years of recurrent hemoptysis or recurrent severe hemoptysis. The actual rate of recurrence was shown on the y-axis, and the nomogram-predicted probability of recurrence was shown on the x-axis

Based on the above independent significant predictors identified by multivariate Cox regression, two nomograms were established to predict the recurrent hemoptysis rate and recurrent severe hemoptysis rate of patients after BAE at 1-year, 3-year, and 6-year time points, respectively (Fig. 4A, B). AUC evaluated the discriminatory performance, which was 0.728 (95% CI, 0.688–0.769) in the recurrent hemoptysis group, and 0.709 (95% CI, 0.653–0.765) in the recurrent severe hemoptysis group (Fig. 4C). Finally, the calibration plot of the nomograms was performed at 2-year timepoint using 1000 bootstrap resamples, which showed good calibration (Fig. 4D).

### Characteristics of abnormal arteries

**Fig. 5:**
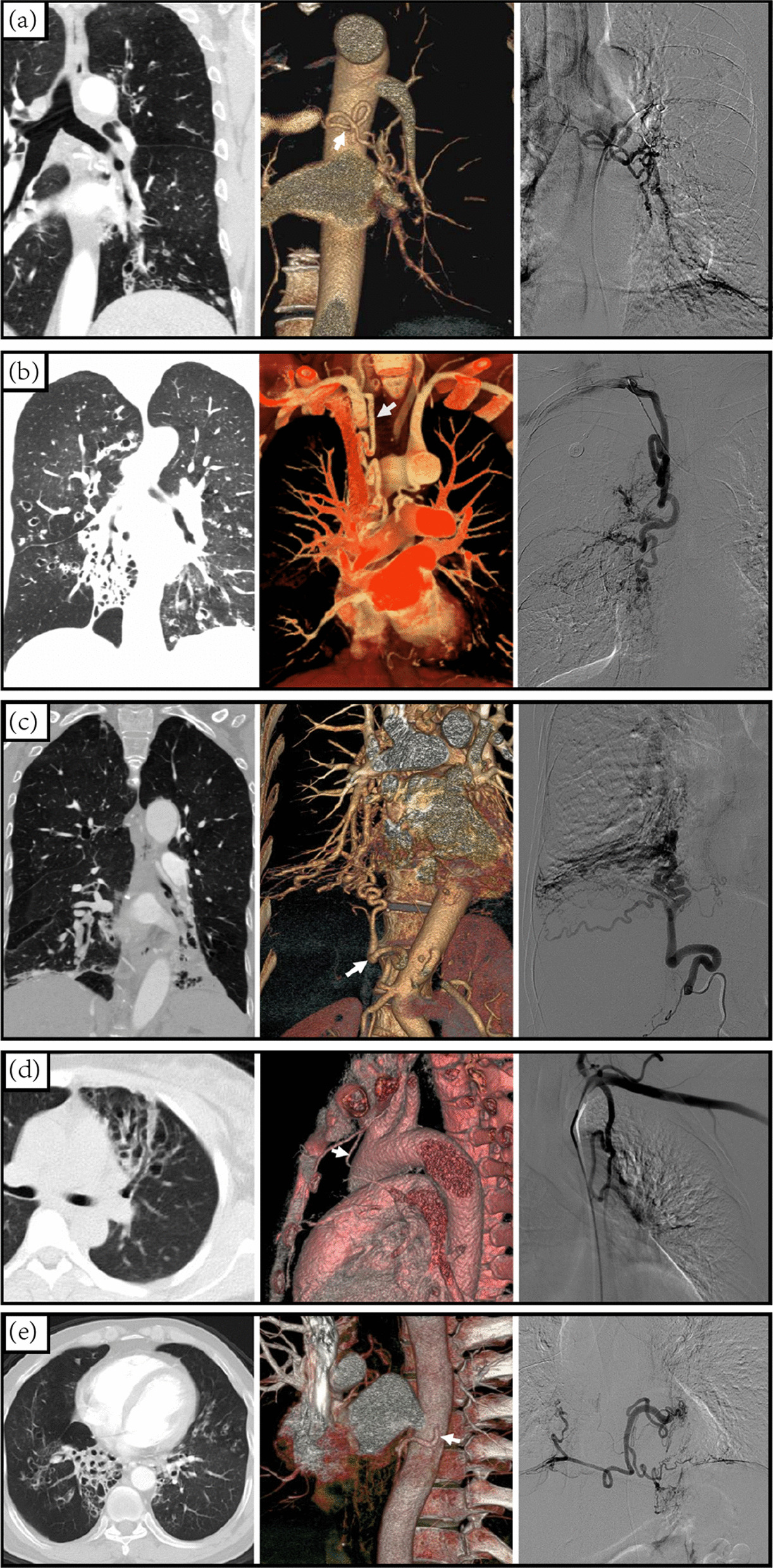
Representative images of BAE for idiopathic bronchiectasis-related hemoptysis with CT (left panel), volume reconstruction (middle panel), and DSA (right panel): (**A**) Left bronchial arteries. (**B**) Aberrant bronchial arteries. (**C**) Right inferior phrenic artery. (**D**) Left internal thoracic artery. (**E**) Proper esophageal artery. CT showed bronchiectasis with mucous embolism. The arrow points to the thickening, engorgement, and tortuosity arteries

Abnormal arteries were found on CTA images of all patients, and almost every patient had abnormal BA. Abnormal left inferior phrenic arteries, the most common abnormal NBSAs, were identified in 208 patients. Abnormal AbBAs on CTA were found in 124 patients (Additional file 3: Figure S3). Half of the abnormal arteries were anatomically normal bronchial arteries, of which 5.29% were abnormal AbBAs on CTA. The other 44.82% were NBSAs. All arteries showed thickening, some showed exudation and vascular network formation, and a few formed systemic-pulmonary artery shunts. Representative images before BAE were shown in Fig. 5.

To further explore the relationship between AbBAs and recurrence, the origination of abnormal AbBAs on CTA was analyzed in detail. AbBAs originating from the subclavian arteries and their branches were the most found in 41 patients (32%). The recurrence rate of hemoptysis patients with abnormal arteries was about 58% (72/124), and the recurrence rate of severe hemoptysis was more than 30% (38/124). Recurrent hemoptysis was found in all patients with AbBAs originating from the proper esophageal artery. More than half of patients with AbBAs originating from the left gastric artery recurred with severe hemoptysis. Patients with AbBAs originating from the left common carotid arteries accounted for only 1.57% (n = 2) of abnormal AbBAs, and no recurrence was observed (Additional file 3: Figure S3).

## Discussion

In this multicenter retrospective cohort study, the presence of recurrent hemoptysis was high in patients with idiopathic bronchiectasis after BAE affecting almost half of patients, while one-quarter of patients needed a second BAE or lobectomy. Recurrent hemoptysis correlated with *P. aeruginosa* infection, massive sputum production, abnormal AbBAs, and extensive bronchiectasis. Furthermore, recurrent severe hemoptysis was associated with *P. aeruginosa* infection, abnormal AbBAs, and age.

Several studies have investigated the recurrence of severe hemoptysis among different aetiologies of bronchiectasis. All 588 patients in this study were idiopathic bronchiectasis patients, and this is the first study to our knowledge assessing a single aetiology on BAE and hemoptysis recurrence. The cumulative recurrent severe hemoptysis rates in this study were similar to previous studies [12, 13, 18]. The recurrence rate of hemoptysis in patients with bronchiectasis was lower than in patients with mycobacterium, fungal infection, or a post-tuberculous phenotype of bronchiectasis [20, 21]. A previous retrospective study of 106 bronchiectasis patients revealed that neither the bacteria, presence of NBSAs, nor the number of bronchiectatic lobes correlated with hemoptysis control [18]. Notably, this previous study [18] did not focus on *P. aeruginosa*, and the number of bronchiectatic lobes was less than that in our study. Another study showed lung destruction, sex, and systemic arterial-pulmonary circulation shunts could be used to predict recurrence [13]. Instead of lung destruction, this study classified and described the CT findings of patients in detail [22].

Patients with *P. aeruginosa* had more severe radiological findings, worse lung function, and more stubborn disease [23–25]. *P. aeruginosa*, as a risk factor for exacerbated bronchiectasis [26], has attracted attention in bronchiectasis because of its high drug resistance, easy colonization, and repeated exacerbation [27–29]. Multiple mutations exist in the genotype of *P. aeruginosa* isolated from patients with bronchiectasis [27]. We have reported that virulence genes exoU or pldA of *P. aeruginosa* led to a higher incidence of exacerbation in patients with idiopathic bronchiectasis [30]. A vicious cycle of inflammation and infection can perpetuate symptoms and further airway damage. Guidelines recommend that patients with first isolation *P. aeruginosa* should be treated with eradication antibiotics [31], while long-term inhaled antibiotic or oral macrolides are recommended for patients with three or more repeated exacerbations per year [14, 32]. Whether long-term antimicrobial prophylaxis (such as macrolide) can reduce the risk of recurrent hemoptysis needs to be confirmed in prospective studies. Furthermore, this study showed that severe recurrent hemoptysis in patients with isolated *P. aeruginosa* was 2.87 times higher than that in patients without *P. aeruginosa*. Bronchiectasis patients with hemoptysis may be treated with antibiotics guided by routine sputum examination and antibiotic susceptibility results. More prospective or even randomized controlled trials are needed to explore management strategies for patients with bronchiectasis with *P. aeruginosa* infection to further optimize treatment guidelines.

Considering that patients with *P. aeruginosa* infection manifested more purulent sputum [33], this study specifically excluded the possibility of collinearity between *P. aeruginosa* and 24-h sputum volume so that sputum volume was an independent predictor of recurrent hemoptysis. Although recommended for bronchiectasis [14], airway clearance techniques might aggravate hemoptysis. Therefore, it is necessary to select appropriate airway clearance techniques or use expectorants regularly to discharge sputum.

In general, the severity of bronchiectasis should be associated with a recurrence incidence of hemoptysis. Therefore, we evaluated the severity of bronchiectasis with the bronchoarterial ratio, the number of bronchiectatic lobes, and bronchiectatic type on CT scans, and explored the correlation of bronchiectasis severity with recurrence. The multiple Cox regression analysis showed that recurrent hemoptysis was positively correlated with the number of bronchiectatic lobes. A Korean study showed that the number of bronchiectatic lobes was positively related to the in‑hospital mortality, and negatively related to the survival rate [34]. Efforts should be made to delay the progression of bronchiectatic lobes, especially in patients with hemoptysis.

Abnormal arteries manifested as dilatation, exudation, vasoganglion formation, aneurysms, and arterial-pulmonary circulation shunts on CTA or digital subtraction angiography. Proper esophageal arteries accounted for 17.7% (n = 183) of NBSAs in this study. We have reported that proper esophageal arteries can be involved in hemoptysis, especially when the basal segment of the lobe is involved [35], which was confirmed in South Korea [36]. The pulmonary plexus originates from the dorsal aorta during embryonic development. Once connected to the pulmonary arteries, the primitive BAs begin to degenerate, which results in only some branches formed in adults [37]. The proportion of AbBAs originating from the subclavian and its branches were the largest, indicating more persistence of primitive branches of high origin [38]. According to embryonic development, AbBAs are also probably potential in the general population; however, these arteries are too thin to be discerned on CTA. Chronic infection may induce vascular thickening, making them easier to identify. Compared to patients without abnormal AbBAs on CTA, patients with abnormal AbBAs had an increased incidence of recurrent severe hemoptysis in this study, supporting the idea that the pulmonary vascular system developed before the tracheal system and the development of BAs was due to some stimulus [37]. As the lung developed, some blood vessels degenerated, but arteries of incomplete degeneration may thicken during infection. Therefore, more attention should be paid to abnormal AbBAs.

One strength of our study is that this was a multicenter study with the largest cohort of patients with idiopathic bronchiectasis. Endovascular treatments of hemoptysis were performed in more than 1000 cases per year in our hospital, and the operators of BAE in the eight centers were trained uniformly, which provided reassurance for this research. The second strength is that specific abnormal arteries were identified. The Third strength is that this study indicated the significance of *P. aeruginosa* in the recurrence of hemoptysis and suggested the importance of *P. aeruginosa* clearance therapy.

This study has some limitations as well. First, there is recall bias and reporting bias. Second, some unspecified and unquantifiable factors, such as environmental exposures, climatic variation, lifestyle, and psychosocial factors, also affect the outcomes of bronchiectasis [39] and require a more thorough investigation. We hope to improve these data in the future by conducting a prospective trial.

In summary, factors associated with relapse of hemoptysis after BAE in patients with idiopathic bronchiectasis are *P. aeruginosa* infection, massive sputum production, abnormal AbBAs, and extensive bronchiectasis. Recurrent severe hemoptysis correlated with age, *P. aeruginosa* infection, and abnormal AbBAs. In view of we could not change the responsible vascular malformation and the degree of bronchiectasis. Therefore, the isolation of *P. aeruginosa* from patients requires further attention, and the appropriate airway clearance techniques should be individually applied after BAE.

## Supplementary Information

**Additional file 1: Figure S1.** The cumulative rates of patients with recurrent hemoptysis-free or recurrent severe hemoptysis-free with *Pseudomonas aeruginosa*, abnormal AbBAs on CTA, extensive bronchiectasis, and high 24-h sputum volume.

**Additional file 2: Figure S2.** Distribution characteristics of abnormal arteries in 588 patients. BAs, bronchial arteries; NBSAs, non-bronchial system arteries.

**Additional file 3: Figure S3.** Specific origination, the recurrence rate, and the severe recurrence rate of abnormal AbBAs on CTA. (A) The proportion of certain abnormal AbBAs on CTA to all abnormal AbBAs on CTA. (B) Ratio of recurrent hemoptysis group to all patients per abnormal AbBAs on CTA. (C) Ratio of recurrent severe hemoptysis group to all patients per abnormal AbBAs on CTA.

**Additional file 4: Table S1.** Univariate Cox regression and multivariate Cox regression analysis of the factors associated with recurrent hemoptysis of patients with idiopathic bronchiectasis after BAE. **Table S2.** Univariate Cox regression and multivariate Cox regression analysis of the factors associated with recurrent severe hemoptysis of patients with idiopathic bronchiectasis after BAE.

## Data Availability

The datasets used and/or analysed during the current study are available from the corresponding author on reasonable request.

## Abbreviations

AbBAs: Aberrant bronchial arteries
AUC: Area under the receiver operating characteristic curve
BAE: Bronchial artery embolization
BAs: Bronchial arteries
CT: Computed tomography
CTA: CT angiography
CI: Confidence intervals
HR: Hazard ratio
IQR: Interquartile range
NBSAs: Non-bronchial systemic arteries
*P. aeruginosa*: *Pseudomonas aeruginosa*
SD: Standard deviation
