# Supplementary material for: Pseudomonas aeruginosa isolation is an important predictor for recurrent hemoptysis after bronchial artery embolization in patients with idiopathic bronchiectasis: a multicenter cohort study
Source: Respir Res. 2023 Mar 18;24:84. doi: 10.1186/s12931-023-02391-9 (PMC10024824; doi:10.1186/s12931-023-02391-9)
Supplement: Supplementary file 1 — Additional file 1: Figure S1. The cumulative rates of patients with recurrent hemoptysis-free or recurrent severe hemoptysis-free with Pseudomonas aeruginosa, abnormal AbBAs on CTA, extensive bronchiectasis, and high 24-h sputum volume. [file 12931_2023_2391_MOESM1_ESM.pdf]

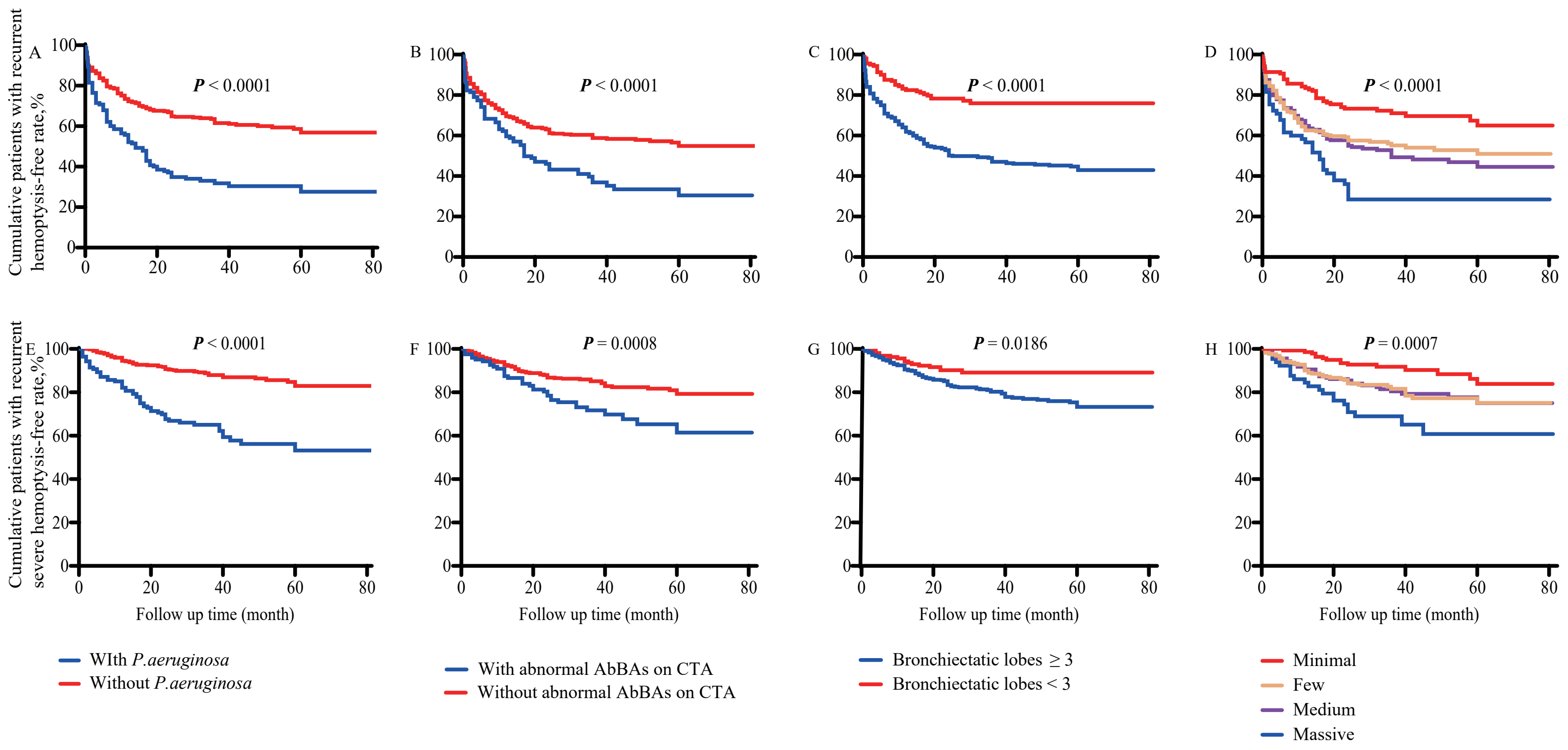

A
B
C
D
E
F
G
H
Cumulative patients with recurrent hemoptysis-free rate,%
Cumulative patients with recurrent severe hemoptysis-free rate,%
P < 0.0001
P < 0.0001
P < 0.0001
P < 0.0001
P < 0.0001
P = 0.0008
P = 0.0186
P = 0.0007
Follow up time (month)
WIth P.aeruginosa
Without P.aeruginosa
With abnormal AbBAs on CTA
Without abnormal AbBAs on CTA
Bronchiectatic lobes ≥ 3
Bronchiectatic lobes < 3
Minimal
Few
Medium
Massive
