# Supplementary figures and images for: Pseudomonas aeruginosa isolation is an important predictor for recurrent hemoptysis after bronchial artery embolization in patients with idiopathic bronchiectasis: a multicenter cohort study
Source: Respir Res. 2023 Mar 18;24:84. doi: 10.1186/s12931-023-02391-9 (PMC10024824; doi:10.1186/s12931-023-02391-9)

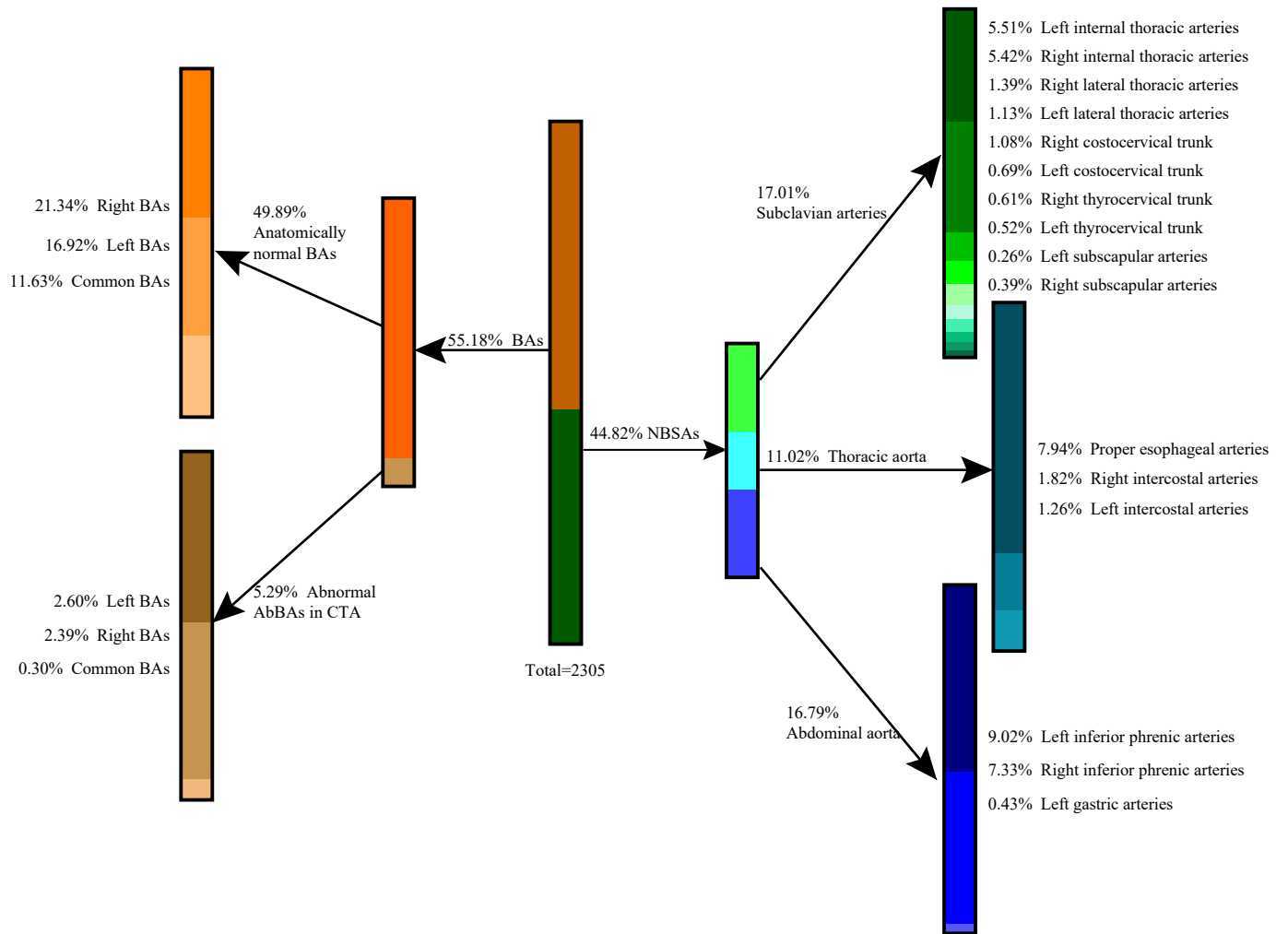

Supplement: Supplementary file 2 — Additional file 2: Figure S2. Distribution characteristics of abnormal arteries in 588 patients. BAs, bronchial arteries; NBSAs, non-bronchial system arteries. [file 12931_2023_2391_MOESM2_ESM.pdf]

A

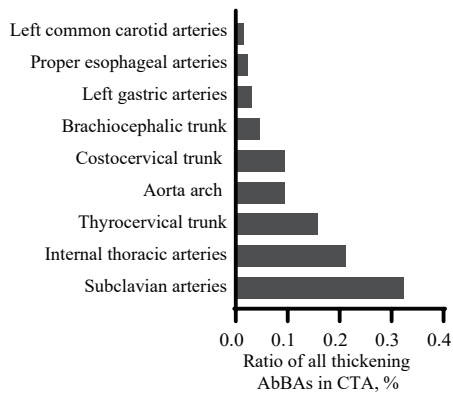

B

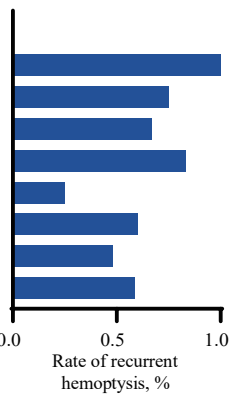

C

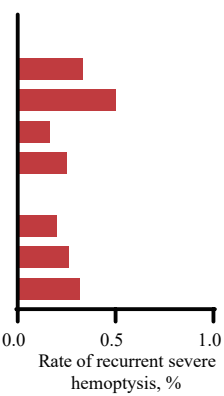

Supplement: Supplementary file 3 — Additional file 3: Figure S3. Specific origination, the recurrence rate, and the severe recurrence rate of abnormal AbBAs on CTA. (A) The proportion of certain abnormal AbBAs on CTA to all abnormal AbBAs on CTA. (B) Ratio of recurrent hemoptysis group to all patients per abnormal AbBAs on CTA. (C) Ratio of recurrent severe hemoptysis group to all patients per abnormal AbBAs on CTA. [file 12931_2023_2391_MOESM3_ESM.pdf]
