# Supplementary material for: Pseudomonas aeruginosa isolation is an important predictor for recurrent hemoptysis after bronchial artery embolization in patients with idiopathic bronchiectasis: a multicenter cohort study
Source: Respir Res. 2023 Mar 18;24:84. doi: 10.1186/s12931-023-02391-9 (PMC10024824; doi:10.1186/s12931-023-02391-9)
Supplement: Supplementary file 4 — Additional file 4: Table S1. Univariate Cox regression and multivariate Cox regression analysis of the factors associated with recurrent hemoptysis of patients with idiopathic bronchiectasis after BAE. Table S2. Univariate Cox regression and multivariate Cox regression analysis of the factors associated with recurrent severe hemoptysis of patients with idiopathic bronchiectasis after BAE. [file 12931_2023_2391_MOESM4_ESM.docx]

**Table S1.** Univariate Cox regression and multivariate Cox regression analysis of the factors associated with recurrent hemoptysis of patients with idiopathic bronchiectasis after BAE.

| **Characteristics** | **Univariate Cox regression** | | **Multivariate Cox regression** | | |
| --- | --- | --- | --- | --- | --- |
|  | **HR (95%CI)** | ***p* Value** | **HR (95%CI)** | ***p* Value** |  |
| Duration of bronchiectasis, years | 1.014(1.006,1.022) | 0.001 |  |  | |
| Duration of hemoptysis, years | 1.013(1.005,1.022) | 0.002 |  |  | |
| 24-h sputum volume^a^  Minimal  Few  Medium  Massive | Ref  1.759(1.225,2.524)  1.977(1.363,2.868)  3.233(2.114,4.945) | <0.001  0.002  <0.001  <0.001 | Ref  1.313(0.899,1.917)  1.562(1.062,2.296)  2.027(1.278,3.215) | 0.015  0.159  0.023  0.003 | |
| Isolation of *Pseudomonas aeruginosa* | 2.207(1.717,2.836) | <0.001 | 1.540(1.157,2.051) | 0.003 | |
| Number of bronchiectatic lobes ≥3 | 2.696(1.895,3.834) | <0.001 | 1.968(1.270,3.047) | 0.002 | |
| Bronchoarterial ratio^b^  1-2 times  2-3 times  >3 times | Ref  1.364(0.958,1.944)  2.195(1.672,2.881) | <0.001  0.085  <0.001 |  |  | |
| Bronchiectatic type  Cylindrical  Cystic  Mixed | Ref  1.199(0.766,1.875)  1.975(1.437,2.714) | <0.001  0.427  <0.001 |  |  | |
| Emphysema | 0.690(0.493,0.966) | 0.031 |  |  | |
| Abnormal AbBAs on CTA | 1.745(1.337,2.277) | <0.001 | 1.429(1.076,1.897) | 0.014 | |
| Inferior phrenic artery | 1.277(1.004,1.624) | 0.046 |  |  | |

HR (95% CI), hazard ratio (95% confidence interval). AbBAs, aberrant BAs; CTA, Computed tomography angiography.

a, Minimal, few, medium, and massive represented sputum volumes of <10 mL, 10-50 mL, 50-100 mL, and≥100 mL, respectively.

b, The ratio of bronchia lumen to vessel diameter.

**Table S2.** Univariate Cox regression and multivariate Cox regression analysis of the factors associated with recurrent severe hemoptysis of patients with idiopathic bronchiectasis after BAE.

| **Characteristics** | **Univariate Cox regression** | | **Multivariate Cox regression** | | |
| --- | --- | --- | --- | --- | --- |
|  | **HR (95%CI)** | ***p* Value** | **HR (95%CI)** | ***p* Value** |  |
| Age, years | 1.020(1.004,1.036) | 0.013 | 1.020(1.003,1.037) | 0.018 | |
| Duration of bronchiectasis, years | 1.021(1.009,1.034) | 0.001 |  |  | |
| Duration of hemoptysis, years | 1.020(1.007,1.033) | 0.002 |  |  | |
| Hemoptysis volume, mL | 1.001(.999,1.003) | 0.268 |  |  | |
| 24-h sputum volume†  Minimal  Few  Medium  Massive | Ref  1.952(1.099,3.466)  2.032(1.121,3.681)  3.573(1.863,6.852) | 0.002  0.022  0.019  <0.001 |  |  | |
| Isolation of *Pseudomonas aeruginosa* | 3.590(2.486,5.185) | <0.001 | 2.869(1.888,4.360) | <0.001 | |
| Number of bronchiectatic lobes ≥3 | 1.923(1.129,3.276) | 0.016 |  |  | |
| Bronchoarterial ratio§  1-2 times  2-3times  >3times | Ref  1.914(1.107,3.309)  2.639(1.698,4.102) | <0.001  0.020  <0.001 |  |  | |
| Bronchiectatic type  Cylindrical  Cystic  Mixed | Ref  1.156(0.561,2.381)  1.757(1.064,2.903) | 0.054  0.695  0.028 |  |  | |
| Atelectasis | 1.620(1.109,2.366) | 0.013 |  |  | |
| Abnormal AbBAs on CTA | 2.024(1.367,2.996) | <0.001 | 1.592(1.052,2.407) | 0.028 | |
| Inferior phrenic artery | 1.483(1.026,2.145) | 0.036 |  |  | |

HR (95% CI), hazard ratio (95% confidence interval). AbBAs, aberrant BAs; CTA, Computed tomography angiography.

†, Minimal, few, medium, and massive represented sputum volumes of <10 mL, 10-50 mL, 50-100 mL, and ≥100 mL, respectively.

§, The ratio of bronchia lumen to vessel diameter.
